# IL-10 Modulates Th17 Pathogenicity during Autoimmune Diseases

**DOI:** 10.4172/2155-9899.1000400

**Published:** 2016-03-22

**Authors:** Beichu Guo

**Affiliations:** 1 Department of Microbiology and Immunology, Medical University of South Carolina (MUSC), Charleston, South Carolina 29425-5040, USA; 2 Hollings Cancer Center, Medical University of South Carolina (MUSC), Charleston, South Carolina 29425-5040, USA

**Keywords:** Autoimmune disease, Th17, IL-10, Inflammation, Multiple sclerosis, Colitis, Interferon, IL-27, IL-1, Innate immunity

## Abstract

The immune system is essential for host defense against pathogen infections; however dysregulated immune response may lead to inflammatory or autoimmune diseases. Elevated activation of both innate immune cells and T cells such as Th17 cells are linked to many autoimmune diseases, including Multiple Sclerosis (MS), arthritis and inflammatory bowel disease (IBD). To keep immune homeostasis, the immune system develops a number of negative feedback mechanisms, such as the production of anti-inflammatory cytokine IL-10, to dampen excessive production of inflammatory cytokines and uncontrolled activation of immune cells. Our recent studies uncover a novel immunoregulatory function of interferon (IFN) pathways on the innate and antigen-specific immune response. Our results show that IFNα/β induced IL-10 production from macrophages and Th17 cells, which in turn negatively regulated Th17 function in autoimmune diseases such as Experimental Allergic Encephalomyelitis (EAE), an animal model of human MS. In a chronic colitis model resembling human IBD, we also found that IL-10 inhibited inflammasome/IL-1 pathway, and the pathogenicity of Th17 cells, leading to reduced chronic intestinal inflammation. Results from our and other studies further suggest that IL-10 produced by both macrophages and regulatory T cells may shift Th17 into more regulatory phenotypes, leading to reduced inflammatory response.

## Introduction

Autoimmune diseases are characterized by dysregulated inflammation and immune response, leading to the loss of tolerance to self-antigens [1–4]. Accumulating evidence suggests that both innate immune system such as Toll-like receptors (TLRs) and adaptive immune system such as T cells contribute to the pathogenesis of inflammatory and autoimmune diseases [5–10]. The innate immune system is not only the first line of defense against microbes in vertebrates, but also critical for the activation and development of adaptive immunity, including various types of T cells [11–15]. While essential for initiating rapid inflammatory response and priming host adaptive immune system, excess or prolonged inflammation is harmful to the host, which can lead to tissue damage and autoimmune diseases [1,5,7–9,16–20]. Recent studies demonstrate that Th17 cells, a subtype of CD4^+^ T helper cells, play a critical role in many autoimmune and inflammatory diseases such as multiple sclerosis (MS), rheumatoid arthritis (RA), psoriasis and inflammatory bowel disease (IBD) [10,21–26]. Patients in those autoimmune diseases often have elevated levels of IL-17A and IL-17F in blood or affected tissues. Therefore, it is critical to understand the mechanisms by which the host immune system constrains Th17 cell and to explore therapeutic strategies to modulate Th17 function in autoimmune and inflammatory diseases. In addition, efforts have been made to develop antibodies that can block IL-17 or cytokines inducing Th17 cell differentiation for the treatment of autoimmune diseases [27–31].

To limit tissue damage, the immune system evolves regulatory mechanisms to control the intensity and duration of immune response. IL-10, produced by innate immune cells as well as T and B cells, functions as one of major negative feedback mechanisms to dampen uncontrolled production of inflammatory cytokines and excessive inflammation [32–40]. This review will highlight our recent findings demonstrating that type I interferon (IFN) pathway plays critical roles in suppressing Th17-associated autoimmune and inflammatory diseases, such as experimental allergic encephalomyelitis (EAE), through the induction of IL-10. We will also briefly discuss the role of IL-10 produced by regulatory T cells in suppression of Th17-mediated inflammation.

### IL-17 and Th17 cells

Recent progress indicates that T cell differentiation exhibits considerable diversity and plasticity depending on inflammatory milieu provided by innate immune cells [7,41–49]. During the differentiation of naive CD4 T cells, Th1 cells are induced by IL-12 and produce large quantities of interferon-γ. Th2 cells secrete IL-4, IL-5 and IL-13. TGFβ and IL-6 induce Th17 cells to produce IL-17A (generally referred to as IL-17 in the literature), IL-17F and IL-22 [8,21,50–53]. Other T help cell subsets such as Th9 and Th22 are also identified though their functions *in vivo* require further study [8,54]. The IL-17 cytokine family has at least six members: IL-17A, IL-17B, IL-17C, IL-17D, IL-17E (also referred as IL-25) and IL-17F. Among the members of this family, IL-17A or IL-17, and IL-17F are mostly studied due to their important functions in immune response and autoimmunity. Even though Th17 cells are a major source of IL-17, other types of cells including CD8 (Tc17), γδT cells, NK cells and innate lymphoid cells are also able to produce IL-17 [55,56]. Experimental and clinical studies have linked abnormal levels of IL-17 to autoimmune diseases such as multiple sclerosis, rheumatoid arthritis, psoriasis [10,21–26]. Furthermore, clinical trials using cytokines to decrease Th17 cells or pharmacological interventions with antibodies targeting IL-17 levels or IL-17R function have been performed to ameliorate autoimmune diseases [2,27–29,57–59].

The differentiation of Th17 cells from naïve CD4 T cells is regulated by a complex network of cytokines and transcriptional factors. Early studies demonstrate that the differentiation of Th17 cells depends on TGFβ and IL-6, which induce naïve T cells to secrete IL-21. IL-21 in turn functions in a positive autocrine loop to upregulate the expression of Th17 lineage-specific transcription factor RORγt and cytokines, whereas IL-23 is responsible for maintenance and expansion of Th17 cell populations [8,60–64]. Subsequent studies found that other cytokines, including IL-1, IL-13, IL-18, IL-22, and transcription factors, including STAT3, Runx1, IRF4, also can influence Th17 differentiation [65–70]. Although there is debate about the role of IL-1β in the differentiation of human or murine Th17 cells, recent studies suggest that IL-1 drives the generation of pathogenic Th17 cells in experimental autoimmune diseases [71–77]. Because of reciprocal nature of T cell differentiation, studies demonstrate that Th1 or Th2 cytokines such as IL-4 and IFNγ can inhibit the development of Th17 cells [42,51,62–64,78]. We and other groups found that type IFNs as well as IL-10 and IL-27 were potent negative regulators of Th17 cells [38–40,79]. Although Th17 cells are classically described as a novel CD4 T cells producing IL-17, accumulating evidence indicates that Th17 cells can exhibit a spectrum of phenotypes and effector functions in response to different inflammatory microenvironments. To better define Th17 cell populations, Peters, Lee and Kuchroo proposed to characterize Th17 cells ranging from classical or more regulated Th17 cells to alternative or more pathogenic Th17 cells [80,81]. In this review, we use similar terms to categorize different Th17 cells, in which IL-10-producing Th17 cells are described as regulatory Th17 cells, whereas pathogenic Th17 cells are generated in the presence of IL-1β or IL-23.

### Innate immunity and autoimmune diseases

The development of Th17 cells and Th17-associated autoimmune diseases are influenced by inflammatory cytokines and self-antigens presented by innate immune cells. A well-studied example is the induction of experimental allergic encephalomyelitis (EAE), an animal model of human MS, which is a chronic autoimmune demyelinating disease in CNS characterized by the infiltration of inflammatory cells, including macrophages and T cells, into the central nervous system [57,82–86]. Previously viewed as a T helper type 1 (Th1) cell-mediated disease, studies conducted during the past decade or so suggest that Th17 cells also play an important role in the pathogenesis of neuroinflammtion [54,71,82,87]. As MS is an autoimmune disease mediated by T cells, it indicates that hosts loss tolerance to the self-antigen myelin. In EAE, the neuronal inflammation and destruction of myelin sheath are induced peripherally by injection of both self-antigen and strong adjuvants, usually killed whole mycobacteria, which potently activate multiple innate immune pathways [38,83,84,88].

The induction of innate immunity is mediated by diverse families of pattern recognition receptors (PRRs) that recognize microbial components termed pathogen associated molecular patterns (PAMPs), which can be viewed as a molecular “signature” of the invading pathogens. Major PRRs include Toll-like receptors (TLRs), NOD-like receptors and RIG-I-like receptors [15,89]. Currently, at least 11 TLRs have been identified in mammals, and each receptor is involved in the recognition of a unique set of PAMPs. TLRs sense invading pathogens by recognizing PAMPs in the extra-cellular milieu or in endosomal membrane compartments of innate immune cells. TLR signaling is transduced through the recruitment of different MyD88 adaptor family members, primarily MyD88 and Toll-interleukin-1 receptor domain-containing adaptor inducing IFN-β (TRIF), leading to distinct host response [11,12,90–93]. For example, TLR2 and 9 in macrophages and DCs mainly induce the production of NF-κB-dependent inflammatory cytokines such as IL-12, IL-6 and IL-23; binding of viral related products such as polyIC to TLR3 triggers type I IFN production through the TRIF-dependent pathway; whereas LPS recognized by TLR4 can activate both pathways.

Various experiments demonstrated that TLR ligands, such as LPS, CpG and PGN, could induce EAE in association with self-antigen [5,38,83,94,95], However, not all TLR agonists are able to induce CNS autoimmune inflammation. Results from our lab and other groups have demonstrated that MyD88 signaling pathway is essential for the induction of EAE, since MyD88 deficient mice are resistant to MOG/CFA-induced EAE. Interestingly, polyIC, a TLR3 ligand, has been shown to inhibit EAE development [94,96–99]. Those results indicate that different TLR signals, through downstream MyD88 or TRIF pathways, may modulate antigen-specific immune response and influence the progression of autoimmune diseases. Our recent studies discover immunoregulatory effects of TLR-mediated IFN pathway on the innate and antigen-specific immune response [38–40].

### Type I IFN induction and signaling pathways

The type I IFN family consists of multiple IFNα members and a single IFNβ. These cytokines bind a common receptor, the type I IFN receptor (IFNAR), expressed on a wide variety of cell types, leading to induction of a large set of genes important for antiviral responses [100–103]. Depending upon the types of the pathogens and cells infected, type I IFN can be induced by multiple innate pathways including TLR, RIG-I and DNA sensors [12,101,104–107]. During the induction of IFN, TANK-binding kinase 1 (TBK1) or inducible IκB kinase (IKKi), two non-canonical members of the IKK family, are essential for activation of interferon regulatory factor 3 and 7 (IRF3/IRF7) which control transcription of type I IFN. While TLR3 and TLR4 utilize the adaptor protein TRIF for IRF3/IRF7 phosphorylation and IFN production, TLR7/8 and TLR9 depends on MyD88 signaling complex for activation of IFNs in plasmacytoid Dendritic cells (pDCs). In addition to TLRs in innate immune cells, intracellular RNA receptors such as RIG-I and MDA-5 can induce type I IFN production through adaptor molecules like CARDIF (also known as IPS-1/MAVS/VISA). Cytoplasmic DNA sensors induce IFN production through signaling molecule STING [12,101,104–111].

In addition to anti-viral response, the type I IFNs has potential immunomodulatory effects on both innate and adaptive immune cells. IFNα/β have been used clinically to treat patients with certain tumors and autoimmune diseases, particularly multiple sclerosis (MS) [100,102,103,112,113]. However, the underlying mechanism of IFN therapeutic effects remains not fully understood. Our recent studies indicate that type I IFN pathways limit neuronal inflammation through suppressing Th17 functions. We found that while MyD88-deficient mice failed to develop EAE, mice deficient for IFN induction or receptor signaling had enhanced development of Th17 cells *in vivo* and develop much more severe EAE. Those results demonstrate a functional role of type I IFN induction pathways in modulating Th17 cells as well as Th17-associated autoimmune diseases [38,39].

### Induction of IL-10 by IFNα/β and IL-27

We further elucidate the mechanisms by which IFNβ induction and signaling pathways suppress Th17-associated inflammatory diseases. Our results indicate that IFNα/β-mediated inhibition of Th17 cell activities through the induction of IL-27 and IL-10 directly or indirectly [38–40,79]. IL-10 is a potent anti-inflammatory cytokine with broad effects on both innate and adaptive immune system [38–40]. IL-10 deficiency in mice leads to increased endotoxemia-associated mortality during microbial infection and increased susceptibility to autoimmune disorders such as colitis and autoimmune encephalomyelitis [32,33,37,114,115]. Our previous studies show that type I IFN (IFNα/β) is required for LPS-induced IL-10 upregulation. In addition, we found that defects in type I IFN production and signaling resulted in significant amplification of LPS-induced pro-inflammatory genes and cytokines in macrophages [40]. Our findings suggest that, in addition to its antiviral functions, the type I IFN induces IL-10 to limit TLR-mediated inflammation.

Experimental results from our laboratory indicate that the induction of IL-27 from macrophages may represent one of mechanisms for the inhibitory effect of IFNα/β [38]. IL-27 is a heterodimeric molecule composed of p28 and Epstein-Barr virus–induced gene 3 (Ebi3) subunits, which have homologies to IL-12p35 and p40 respectively. It belongs to IL-12 cytokine family, which also includes IL-12 (p35/p40), IL-23 (p19/p40) and IL-35 (p35/Ebi3) [116–120]. IL-27R deficient mice develop severe immunopathology in several infection and autoimmune models because of excessive inflammation [121–123]. IL-27 is produced by innate immune cells and has various effects on T cell immunity. Of note, even as IL-27 generally suppresses inflammation during infection or autoimmune diseases, a number of studies show that it can promote inflammation [124–129]. Our results show that IL-27 can exert a strong inhibitory effect on the differentiation of Th17 cells. We further demonstrate administration of IL-27 *in vivo* can effectively reduce the symptoms of EAE [38,39]. Although it has been suggested that IL-27 may inhibit Th17 differentiation directly *via* activation of STATs, experimental results from our laboratory and others have demonstrated that IL-27-induced IL-10 may contribute to its immunosuppressive effects [118,125,130]. This suggests that IL-27 can utilize multiple mechanisms to regulate the function of Th17 and other cells during immune responses. However, the relative contribution of IL-10 and IL-27 in suppressing Th17-mediated autoimmunity *in vivo* remains not clear.

### IL-10-mediated modulation of Th17 cells in autoimmune diseases

**Figure 1:**
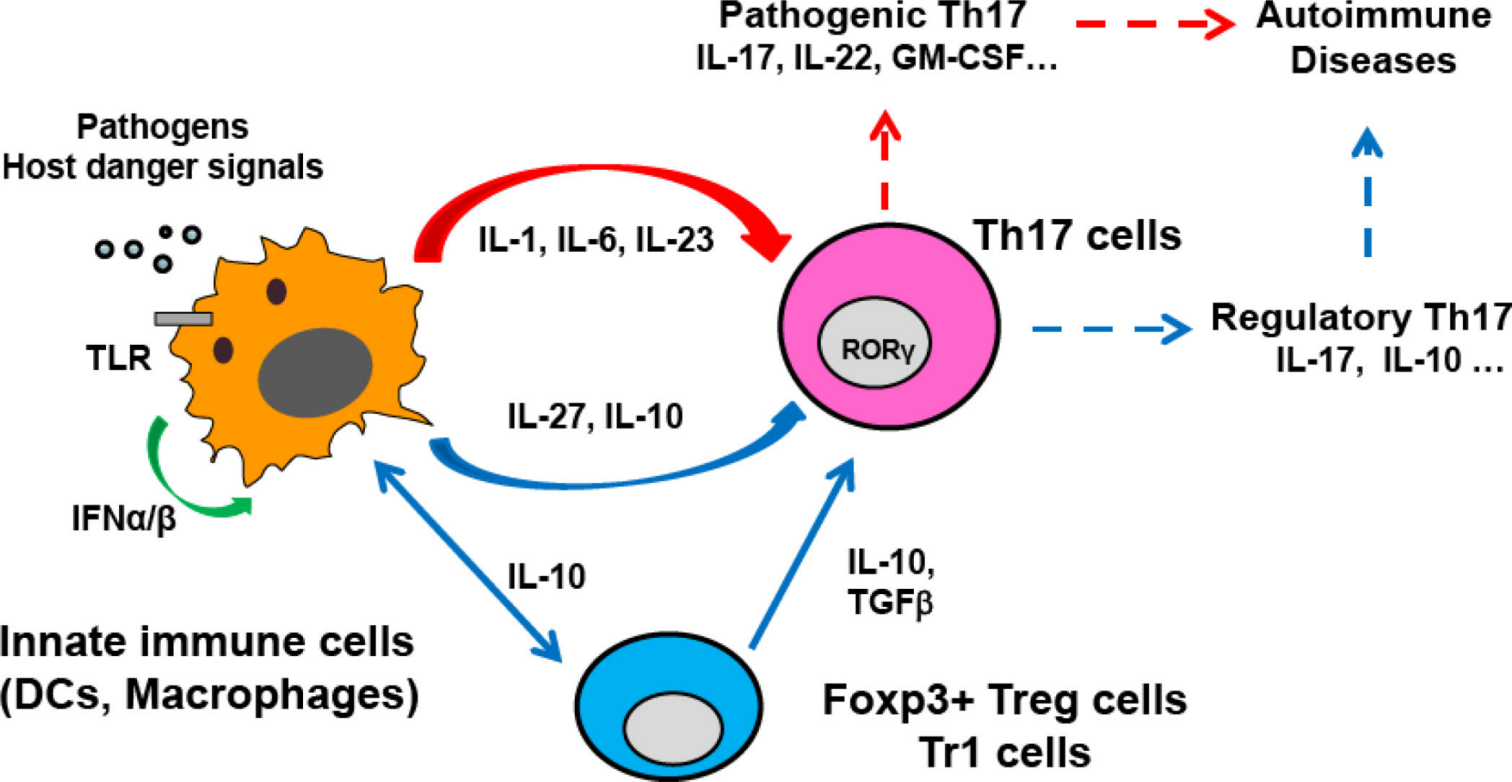
Modulation of Th17 differentiation by IL-10 from innate cells and regulatory T cells Upon encountering pathogens or endogenous danger signals, innate immune cells induce the differentiation of naïve T cells into different T helper subsets including Th17 cells. The phenotypes and function of Th17 cells are influenced by inflammatory cytokine milieu and tailored to the nature of a particular immune response. Inflammatory cytokines such as IL-1β and IL-23 may shift Th17 into more pathogenic types with increased production of GM-CSF and IFNγ. The type I IFN may upregulate anti-inflammatory cytokines such as IL-10 and IL-27 as a negative feedback mechanism to damp uncontrolled production of inflammatory cytokines in both innate and adaptive systems, thereby constraining the development of Th17 cells and excessive inflammation in autoimmune conditions. IL-10 produced by both macrophages and regulatory T cells promote the generation of Th17 cells with regulatory phenotypes.

Emerging evidence points to the heterogeneity and plasticity of Th17 cells [48,49]. However, it remains unknown if self-reactive Th17 cells can be reprogrammed to have less pathogenic activities or even have regulatory effects through modulation of innate pathways. McGeachy et al. reported that Th17 cells cultured with TGFβ and IL-6 led to the generation of IL-10^+^ and IL-17^+^IL-10^+^ cells, which inhibited the pathogenic potential of Th17 cells and suppress the development of EAE in an adoptive transfer EAE experiment. The authors also indicate that IL-10-producing Th17 cells exerted bystander suppressive effects to inhibit fully differentiated pathogenic Th17 populations, and the development of neuronal inflammation [131]. Zielinski et al. also reported that IL-10 production by human Th17 cells in response to pathogens was influenced by IL-1β [73]. Lee et al., through analyzing the induction and molecular signature of pathogenic Th17 cells, proposed that TGFβ and IL-23 serve as major factors in directing the Th17 cells towards either classical phenotype with IL-10 production or alternative (more pathogenic) phenotype with IL-22, GM-CSF and IFNγ expression, respectively [80,81] (Figure 1). Our data suggest that IL-10 production from T cells, possibly Th17cells, may act as a negative regulator to dampen inflammatory response mediated by antigen specific T cells or self-reactive T cells during autoimmune diseases [38,39]. Our recent studies show that T cells in Th17 differentiation conditions treated with IFNβ have reduced expression of IL-17 and RORγt, but increased number of IL-17^+^IL-10^+^ T cells, and enhanced IL-10 production. We also found that IFNβ induced IL-10 production and reduced IL-17 production in antigen specific T cells from EAE mice. Furthermore, treatment of myelin-specific T cells with IFNβ reduces their pathogenic potential, and causes less severity of EAE in an adoptive transfer model. Our data suggest that, in addition to indirectly inhibiting Th17 cells *via* induction of IL-10 and IL-27 from macrophage, IFN-mediated IL-10 production from T cells could contribute to its inhibitory effects on EAE development [38,39]. Results from those studies imply that IL-10 produced by antigen-specific T cells can negatively regulate the pathogenic potential of Th17 cells in inflammatory and autoimmune response.

The impact of IL-10 on Th17 cell function is also illustrated in a chronic colitis model. IL-10 deficient mice spontaneously develop colitis resembling the pathogenesis of human IBD. Furthermore IL-10 or IL-10R mutations are associated with the increased risk of developing colitis in human IBD patients [32,33,37,114,115]. Our recent studies show increased IL-1β production and enhanced inflammasome activities in colon tissues from IL-10 deficient mice compared with that of wild type mice. We also found that intestinal tissues with chronic inflammation produced more IL-17. Furthermore, we provided evidence showing that IL-1β could induce IL-23 and IL-17 from inflamed colon tissues. We demonstrate that inhibition of inflammasome activities with IL-10 or caspase inhibitors could suppress IL-17 production and intestinal inflammation [132]. Our results suggest that chronic activation of inflammasome and production of IL-1β may promote the generation of pathogenic Th17 cells and induce prolonged intestinal inflammation, eventually leading to the development of colitis. While IL-1β has been suggested to induce pathogenic Th17 cells, IL-10, either directly or indirectly *via* macrophages, reduces pathogenicity of Th17 cells or even confers Th17 cell with regulatory functions. However, it is unclear whether IL-10 changes Th17 differentiation programs or IL-10 shifts pathogenicity of already differentiated Th17 *in vivo* during immune response or autoimmune disease conditions.

### IL-10 production and signaling in regulatory T cells

In addition to Th17 cells, various types of T cells, including Th1, Th2, Treg, Tr1 cells as well as CD8 T cells can produce IL-10. In fact, IL-10 was originally identified as a cytokine secreted by Th2 cells [133]. Nevertheless, regulatory T cells, including both Foxp3^+^ Tregs and Tr1 cells, represent a major source of IL-10 produced by T cells [134–140]. Although multiple mechanisms are utilized by regulatory T cells to exert immunosuppressive function, IL-10 derived from Tregs plays a critical role in maintaining immune homeostasis [134–136,141–143]. Therefore, this review will highlight some of studies linking IL-10 production or IL-10 signaling in regulatory T cells to the suppression of Th17-mediated inflammation.

While numerous cell types produce IL-10, different cells may play non-redundant roles as source of IL-10 in the regulation of immune homeostasis. For example, Hadis et al. show that IL-10 produced by CX3CR1-expressing macrophages is essential for the expansion of Treg cells in the intestine system [144]. Murai et al. also demonstrate that that IL-10 released from myeloid cells acts in a paracrine manner on regulatory T cells to maintain Foxp3 expression and immune suppressive function [36]. However, other studies show that IL-10 produced by T regulatory cells induces the anti-inflammatory function of macrophages and intestinal tolerance. Zigmond et al. and Shouval et al. reported that specific IL-10R deficiency, but not IL-10 production deficiency in macrophages led to spontaneous intestinal inflammation [145,146]. Those studies suggest that IL-10R signaling in innate immune cells is critical for the induction of anti-inflammatory function in macrophages, and immune homeostasis in the intestine of mice and humans.

Such conclusion is also supported by previous studies. Roers et al. found that CD4^+^ T cell-specific deletion of IL-10 resulted in development of spontaneous colitis [147], resembling the phenotype in complete IL-10 deficiency. Recent studies demonstrate that IL-10 secreted by Tregs constrains Th17 differentiation and function, thereby, plays a critical role in maintaining immune homeostasis. Rubtsov et al. show that IL-10 produced by Treg cells is not required for suppress systemic autoimmunity, but is critical for constraining inflammation at environmental interfaces where immune cells directly interact with microbes [137]. They found that Treg cell-specific knockout of IL-10 not only resulted in spontaneous inflammation in the colon, but also led to immunological hyperreactivity in the lungs and skin [137]. This study indicates that distinct suppressive mechanisms such as IL-10 production may have specific roles depending on inflammatory sites and response. Furthermore, Chaudhry et al. show that IL-10R signaling in regulatory T cells is important for suppressing inflammation induced by Th17 cells [148]. This elegant study revealed that Treg cell-specific deletion of IL-10R resulted in severe immunemediate colitis, indicating that IL-10R-deficient Tregs loss the ability to inhibit pathogenic Th17 cell responses. Interestingly, IL-10R signaling can further promote IL-10 production from regulatory T cells [148]. Those results imply that regulatory T cells can suppress Th17-mediated inflammation through amplifying a negative regulatory loop of IL-10 production and IL-10R signaling. It also raises the possibility that regulatory T cells may integrate and amplify the anti-inflammatory effects of IL-10 produced by other immune cells including macrophages and various Th cells.

Tr1 cells are Foxp3-negative regulatory T cells that can produce high levels of IL-10 [142,149]. Innate immune cell-derived IL-10, IL-27 and IFN have been shown to induce the differentiation of Tr1 cells [118,139,142,150–153]. While production of IL-10 is a major mechanism by which Tr1 cells suppress inflammation induced by T cells and innate immune cells, Tr1 cells can inhibit T cell immune response through direct cell-cell contact by expressing cytotoxic T-Lymphocyte antigen 4 (CTLA-4) and programmed cell death protein 1 (PD-1) [139,142,154], known as inhibitory receptors or immune checkpoint molecules. Even though Tr1 cells have been identified almost two decades ago for their roles in the suppression of immune response, the signaling and transcriptional programs leading to the generation of Tr1 cells as well as the relationship between Foxp3^+^Treg and Tr1 cells in normal and disease conditions remain poorly understood.

## Future Perspective

It has become widely accepted that inflammation is associated with a variety of human diseases including cancer, colitis, atherosclerosis, and neurodegenerative diseases. Therefore, it is critical to understand how the induction of inflammatory cytokines and cells are regulated. Our recent studies uncover novel immunoregulatory function of IFN during immune response. Our results also establish causal links between IFNα/β and the induction of IL-27 and IL-10 in the inhibition of Th17-mediated inflammation. Although enormous progress has been made in understanding Th17 phenotypes and differentiation in cell culture system, the contribution of various innate pathways and cytokines to the differentiation and function of Th17 cells *in vivo* is still poorly understood. Whether IL-1 or IL-10 signals generate distinct Th17 phenotypes or such phenotypic difference translates into classic/regulatory versus pathogenic Th17 cells needs to be further tested in autoimmune diseases models. It should be emphasized that whether Th17 cells are beneficial or pathogenic to a host depends on the nature of immune response and the context of diseases. In certain autoimmune diseases such as MS, RA and colitis, the inflammatory symptoms could be ameliorated by treatments with IL-1β or IL-17 blockade. However, in some other diseases such as infections and tumors, it is speculated that IL-10-porducing or regulatory Th17 cells may inhibit host immune response, leading to persistent/chronic infections or reduced anti-tumor immunity. In contrast, alternative Th17 cells producing both IL-17 and IFNγ may boost host immune response against tumors [155].
